# Novasomes as Nano-Vesicular Carriers to Enhance Topical Delivery of Fluconazole: A New Approach to Treat Fungal Infections

**DOI:** 10.3390/molecules27092936

**Published:** 2022-05-04

**Authors:** Iman Fatima, Akhtar Rasul, Shahid Shah, Malik Saadullah, Nayyer Islam, Ahmed Khames, Ahmad Salawi, Muhammad Masood Ahmed, Yosif Almoshari, Ghulam Abbas, Mohammed A. S. Abourehab, Sajid Mehmood Khan, Zunera Chauhdary, Meshal Alshamrani, Nader Ibrahim Namazi, Demiana M. Naguib

**Affiliations:** 1 Department of Pharmaceutics, Faculty of Pharmaceutical Sciences, Government College University Faisalabad, Faisalabad 38000, Pakistan; iman.mehmood1994@gmail.com (I.F.); pharmaexpert@yahoo.com (N.I.); 2 Department of Pharmacy Practice, Faculty of Pharmaceutical Sciences, Government College University Faisalabad, Faisalabad 38000, Pakistan; shahid.shah@gcuf.edu.pk; 3 Department of Pharmaceutical Chemistry, Faculty of Pharmaceutical Sciences, Government College University Faisalabad, Faisalabad 38000, Pakistan; maliksaadullah@gcuf.edu.pk; 4 Department of Pharmaceutics and Industrial Pharmacy, College of Pharmacy, Taif University, Taif 21944, Saudi Arabia; a.khamies@tu.edu.sa; 5 Department of Pharmaceutics, College of Pharmacy, Jazan University, Jazan 45142, Saudi Arabia; asalawi@jazanu.edu.sa (A.S.); yalmoshari@jazanu.edu.sa (Y.A.); malshamrani@jazanu.edu.sa (M.A.); 6 Faculty of Pharmacy, Bahauddin Zakariya University Multan, Multan 68000, Pakistan; masoodkarni@yahoo.com; 7 Department of Pharmaceutics, Faculty of Pharmacy, Umm Al-Qura University, Makkah 21955, Saudi Arabia; maabourehab@uqu.edu.sa; 8 Department of Pharmaceutics and Industrial Pharmacy, College of Pharmacy, Minia University, Minia 61519, Egypt; 9 Faculty of Pharmacy and Alternative Medicine, The Islamia University Bahawalpur, Bahawalpur 63100, Pakistan; smk.rao83@gmail.com; 10 Department of Pharmacology, Faculty of Pharmaceutical Sciences, Government College University Faisalabad, Faisalabad 38000, Pakistan; zunerach@gcuf.edu.pk; 11 Pharmaceutics and Pharmaceutical Technology Department, College of Pharmacy, Taibah University, Al Madinah Al Munawarah 30001, Saudi Arabia; nnamazi@taibah.edu.sa; 12 Department of Pharmaceutics, Faculty of Pharmacy, Nahda University (NUB), Beni-Suef 62511, Egypt; demiana.monier@nub.edu.eg

**Keywords:** fluconazole, novasomes, ethanol injection technique, antifungal activity, toxicity study

## Abstract

The occurrence of fungal infections has increased over the past two decades. It is observed that superficial fungal infections are treated by conventional dosage forms, which are incapable of treating deep infections due to the barrier activity possessed by the stratum corneum of the skin. This is why the need for a topical preparation with advanced penetration techniques has arisen. This research aimed to encapsulate fluconazole (FLZ) in a novasome in order to improve the topical delivery. The novasomes were prepared using the ethanol injection technique and characterized for percent entrapment efficiency (EE), particle size (PS), zeta potential (ZP), drug release, Fourier-transform infrared spectroscopy (FTIR), differential scanning calorimetry (DSC), thermogravimetric analysis (TGA), scanning electron microscopy (SEM) and antifungal activity. The FN7 formulation with 94.45% EE, 110 nm PS and −24 ZP proved to be the best formulation. The FN7 formulation showed a 96% release of FLZ in 8 h. FTIR showed the compatibility of FLZ with excipients and DSC studies confirmed the thermal stability of FLZ in the developed formulation. The FN7 formulation showed superior inhibition of the growth of *Candida albicans* compared to the FLZ suspension using a resazurin reduction assay, suggesting high efficacy in inhibiting fungal growth.

## 1. Introduction

The occurrence of fungal infections, either superficial or systemic, is reported to be increasing, affecting almost 40 million people across the world [1]. Among the pathogens that cause skin damage, fungal infections are the most common. The strategies used for the treatment of fungal infections are either systemic or topical. The treatment of fungal infections through the conventional systemic route is not preferred due to a number of associated risks, including specific organic toxicity, drug accumulation, drug–drug interaction, drying of the skin and higher medical costs [2]. Topical and transdermal routes provide ease of application and patient compliance, and thus are preferred over systemic routes. However, conventional topical formulations such as creams, powders, and gels are unable to treat skin fungal infections, particularly deep-seated infections, because of their limited capacity to penetrate and permeate the skin [1]. In addition, these formulations are often designed for the immediate release of the drug and can stimulate the body’s immune system, resulting in serious allergic reactions. Therefore, there is a need to design a drug delivery system that not only possesses the nanoparticulate of the drug, but can also exhibit a sustained release pattern. Vesicular systems, in this regard, are the most frequently used systems. Liposomes and niosomes are the traditional vesicular systems, with the disadvantages of a lack of capacity to deliver the drug to the skin or to effectively distribute the drug [3]. Transferosomes and ethosomes are other vesicular systems that have been designed to improve drug delivery to the skin [4].

FLZ is a bis-triazole compound, belonging to the azole class of antifungals, with novel pharmacokinetic properties including stability and relatively high-water solubility, which improves its therapeutic activity. It is used as an antifungal drug for a range of fungal and yeast infections (candidiasis, blastomycosis, coccidiomycosis, cryptococcosis, histoplasmosis, dermatophytosis and pityriasis versicolor). It binds to fungal cytochrome P450 and ceases the conversion of lanosterol to ergosterol, and thus disrupts the fungal membrane. However, when FLZ is applied topically, it causes skin irritation and often leads to systemic absorption, resulting in a failure to achieve antifungal activity. To overcome the obstacles related to the topical delivery of FLZ, a novel drug delivery system is needed.

Novasomes technology is an effective encapsulation process developed by the IGI laboratories to solve the effectiveness and efficiency-related issues with already available drug delivery systems. In general, novasomes are enhanced liposomal and niosomal structures and are prepared using different concentrations of cholesterol with free fatty acids (FFAs) and a monoester of polyoxyethylene fatty acid. They are spherical in shape, with a diameter of 0.1–1.0 microns, comprising between two and seven bilayer membranes with a high-capacity central core to deliver large amounts of hydrophobic and hydrophilic drug substances [5].

The aim of this paper was to investigate the significance of the formulation of FLZ as novasomes. However, the pre-eminence of novasomes over other nano systems in topical delivery is yet to be investigated. Hence, the aim of this study was to explore the capability of novasomes in enhancing the skin penetration of FLZ. To achieve this objective, novasomes were first prepared using the injection method and then subjected to characterization regarding entrapment efficiency (EE), particle size (PS), polydispersity index (PDI) and zeta potential (ZP), and then the optimum formula was selected. The prepared novasomes along with excipients were further characterized by Fourier-transform infrared spectroscopy (FTIR), differential scanning calorimetry (DSC), thermogravimetric analysis (TGA) and scanning electron microscopy (SEM). Moreover, the inhibition efficacy of FLZ for optimum formula in relation to FLZ suspension against *Candida albicans* was determined through resazurin reduction assay. The cytotoxicity studies of novasomes were conducted on albino rats for a period of 90 days.

## 2. Results and Discussion

### 2.1. Preparation of Novasomes

To formulate the novasomes with the desired characteristics, 10 formulations with different types and ratios of surface-active agents (SAAs) and FFAs and the same cholesterol concentrations were prepared. Two formulations were prepared using Span 80 as an SAA and oleic acid as an FFA at different ratios (FN1, 2:1 and FN2, 1:1) using 30 mg cholesterol. The prepared novasomes of FN1 and FN2 failed to show the desired stability and other characteristics. Two more formulations of FN3 and FN4 were prepared using a different FFA (stearic acid), again using two different SSA and FFA ratios and the same concentration of cholesterol for both formulations (30 mg). FN5 and FN6 were prepared using different ratios of Tween 80 and oleic acid (2:1 and 1:1, respectively), but the cholesterol concentration was kept the same (30 mg) for both formulations. FN7 was prepared using Span 60 and oleic acid in a ratio of 2:1 and using 30 mg of cholesterol. Similarly, FN8 was prepared, but the ratios of Span 60 and oleic acid were changed. FN9 and FN10 were prepared using a combination of Span 60 and stearic acid at ratios of 2:1 and 1:1, respectively. The concentration of cholesterol was kept the same for the preparations of FN8, FN9 and FN10. In the presence of Span 60, the novasomes were more stable during storage compared to the other surfactants utilized. In the presence of Span 60 and oleic acid, the prepared novasome had a gel-like consistency. Due to this gel-like consistency, the topical application of the prepared novasome was very easy. All of the aforementioned formulations showed some problems regarding stability, but the FN7 formulation showed stability as well as all of the other desired characteristics, and so was used for the next characterization.

### 2.2. Particle Size and Zeta Potential Analysis

**Figure 1:**
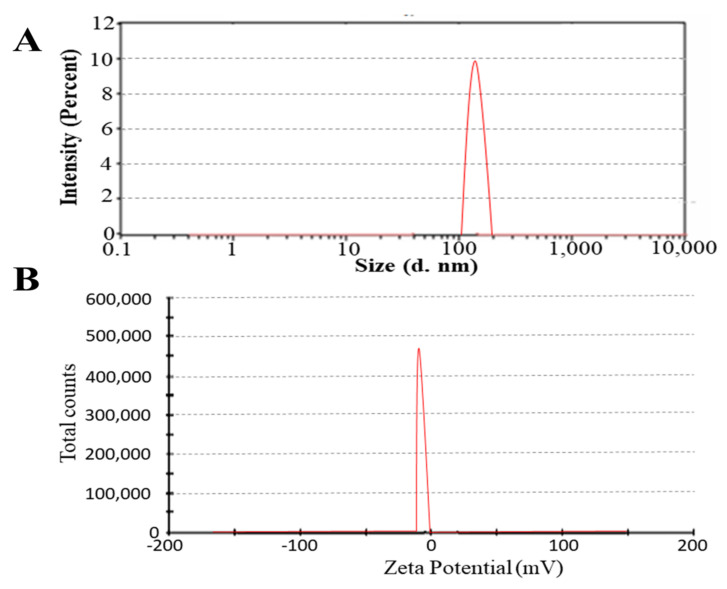
PS (**A**) and ZP (**B**) of optimum formulation FN7 showed a particle size of 110 nm and a ZP value of −24.

The average globule diameter of FN7 was 110 nm, as shown in Figure 1. The polydispersity index (PDI) of all prepared formulations was less than 0.4, as shown in Table 1. The stability of the novasomes depends on the ZP, as it opposes aggregation [6]. The ZP of all of the prepared novasome formulations ranged from −24 to −18, and among these, FN7 was the most stable. Formulations containing SAAs and FFAs in a 2:1 ratio showed more negative ZP values compared to those at 1:1. A more negative value of ZP opposes the aggregation of particles, which leads to the stability of the novasomes.

### 2.3. Entrapment Efficiency (EE)

The EE of all of the formulations ranged from 45.09 to 94.45%, as presented in Table 1. The lowest EE of 45.09% was shown by the FN5 formulation, while FN7 showed the highest EE value of 94.45% [7]. It can be seen that the formulations with Tween 80 as the SAA and the formulations in which stearic acid was used with a combination of any surfactant showed lower EE, but the formulation with oleic acid and Span 60 exhibited a relatively higher EE. Similar findings for the particle size and zeta potential were observed by Housiny et al. when they prepared FLZ-loaded solid lipid nanoparticles for the treatment of pityriasis versicolor [8].

### 2.4. In Vitro Drug Release

**Figure 2:**
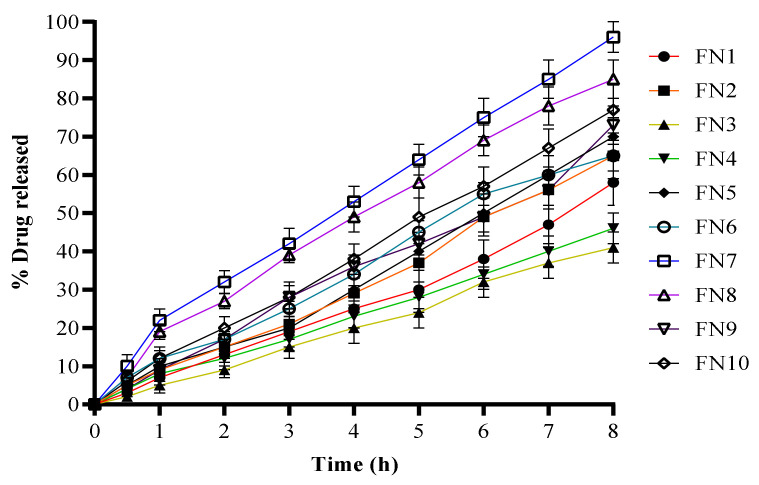
The release of FLZ from prepared novasomes containing different ratios of FFAs and SAAs.

The in vitro drug release from the FLZ novasomes was performed at a physiological temperature in PBS, pH 7.4, at 37 °C using the dialysis bag technique in which the dialysis membrane was presoaked overnight [9]. Figure 2 describes the release profiles of the different FLZ-loaded novasomes. The maximum release of FN1 and FN2 in 8 h was 58% and 65%, respectively. FN3 showed a maximum release of 41% in the 8 h of study. FN4 and FN5 gave a maximum release of 46% and 70%, respectively, over the 8 h period. The maximum release of FN6 was 65%, while FN7 showed 96% of release over 8 h. FN8, FN9 and FN10 gave a maximum release of 85%, 73% and 77%, respectively. Formulations containing Span 60, i.e., FN7, FN8, FN9 and FN10, showed better release than the formulations containing Span 80 and Tween 80, i.e., FN1, FN2, FN3, FN4, FN5 and FN6, as shown in Figure 2. Similarly, it can be seen that formulations with oleic acid showed better release than the formulations with stearic acid. FN7 with both Span 60 and oleic acid in a ratio of 2:2 and 1:1 gave highest maximum release in the 8 h study, showing 96% release with PBS pH 7.4.

### 2.5. Release Kinetics

The value of R^2^ for the zero order ranged from 0.9789 to 0.9984, and for the first order ranged from 0.9439 to 0.9898, indicating that the developed novasome formulations showed zero-order release kinetics. The results suggest the sustained release pattern of FLZ from the novasomes. The value of R^2^ for the Higuchi model ranged from 0.8285 to 0.9356, while the value of the R^2^ of the Hixon–Crowell Model ranged from 0.9612 to 0.9938. The value of n ranged from 1.066 to 1.152, indicating that the formulation followed the non-Fickian release pattern, as shown in Table 2.

### 2.6. Fourier-Transform Infrared Spectroscopy (FTIR)

**Figure 3:**
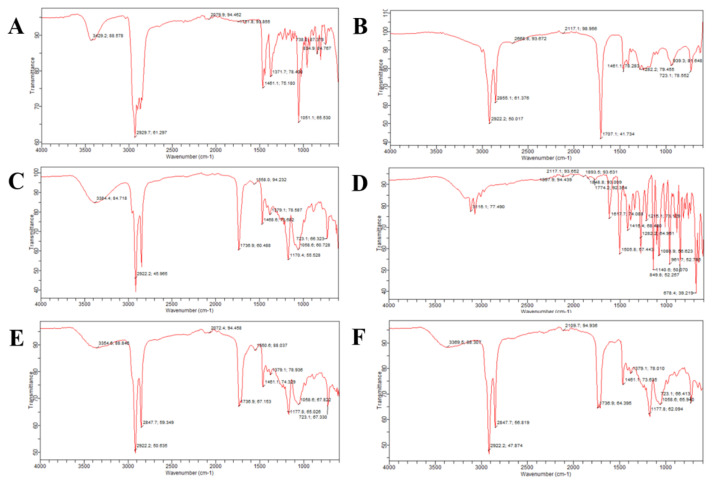
FTIR spectra of cholesterol (**A**), oleic acid (**B**), Span 60 (**C**), FLZ (**D**), drug-unloaded FN7 (**E**) and FLZ-loaded FN7 (**F**).

The stretching of the -OH cholesterol group was observed at 3429 cm^−1^. A symmetrical stretching of the C–H bond of the cholesterol was observed at 2929 cm^−1^, as shown in Figure 3. The oleic acid [10] indicated asymmetrical and symmetric stretching of the -CH_2_ group at 2922 cm^−1^ and 2855 cm^−1^. Span 60 showed O–H stretches at 3384 cm^−1^, aliphatic C–H stretching at 2922 cm^−1^ and stretching of the -CH_3_ group at 1468 cm^−1^. The peak of the cyclic five-membered ring in Span 60 was observed at 1736 cm^−1^. FLZ spectra showed -OH stretching [11] at 3116 cm^−1^, as shown in Figure 3. The peak of the triazole ring was observed at 1617 cm^−1^, -CH_3_ bending was observed at 1416 cm^−1^ and the bending vibration of the -CH_2_ group was observed at 1282 cm^−1^. The FTIR of the unloaded novasomes only showed the peaks of the excipients, but the FTIR of the loaded novasomes showed the peaks excipients as well as the FLZ, indicating that the drug and the polymers used are compatible.

### 2.7. Differential Scanning Calorimetry (DSC)

**Figure 4:**
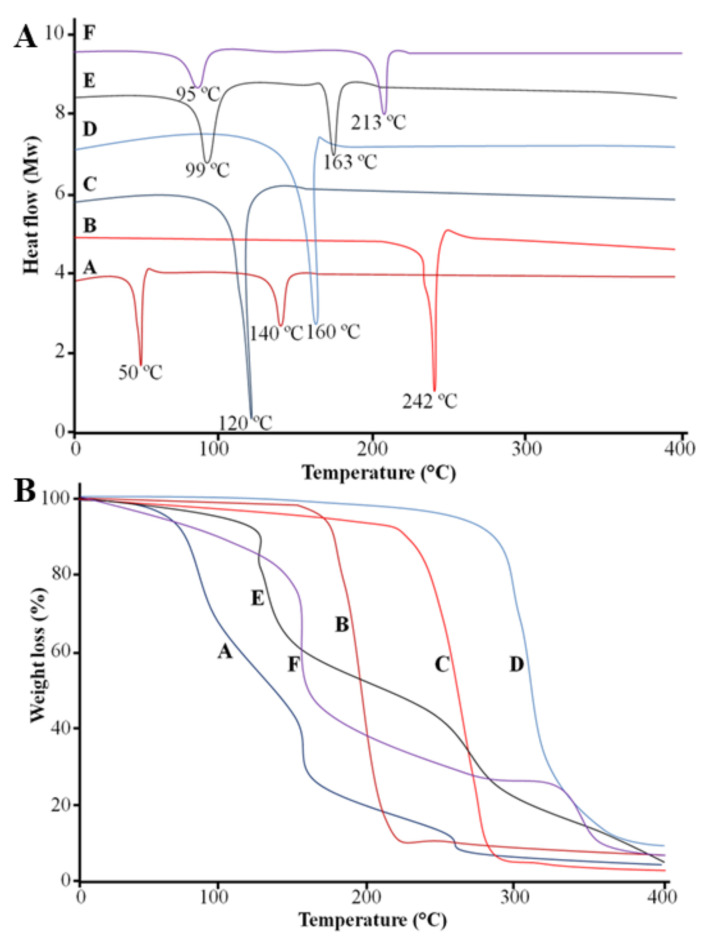
DSC thermograms (**A**) and TGA (**B**) of FLZ (A), oleic acid (B), Span 60 (C), cholesterol (D), blank formulation (E) and FLZ-loaded formulation (F).

The DSC thermogram of the FLZ showed two endothermic peaks at 40 °C and 139 °C, and oleic acid showed an endothermic peak 242 °C, as shown in Figure 4A. One endothermic peak at 120 °C was observed in Span 60, and the cholesterol showed an endothermic peak at 160 °C. The drug-unloaded formulation showed two endothermic peaks at 99 °C and 163 °C, which corresponded to the peaks of the excipients utilized in the formulation. The FLZ-loaded formulation FN7 showed two endothermic peaks at 95 °C and 213 °C, which indicates that the drug and excipients were uniformly distributed and our prepared formulation was stable.

### 2.8. Thermogravimetric Analysis (TGA)

The TGA thermogram of the FLZ showed that weight loss started at 79 °C and continued to 400 °C. The weight loss of the oleic acid was observed at 210 °C. A rapid weight loss of oleic acid was observed between 220 °C and 290 °C. The weight loss of Span 60 started at 237 °C and continued to 400 °C. The weight loss of the cholesterol started at 290 °C. The TGA of the drug-unloaded formulation showed weight loss at 110 °C, and the loaded formulation showed weight loss starting at 100 °C and continuing to 400 °C, which demonstrated the thermostability of the formulation, as shown in Figure 4B.

### 2.9. Scanning Electron Microscopy (SEM)

**Figure 5:**
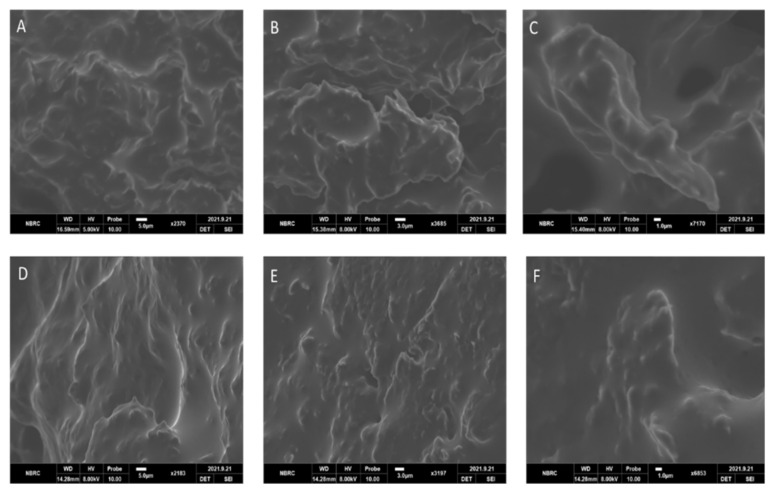
SEM images of drug-unloaded (**A**–**C**) and FLZ-loaded (**D**–**F**) formulations at different resolutions.

The SEM of the unloaded formulations of novasomes showed a cross-linked network structure, as shown in Figure 5A–C. The drug-loaded novasomes also showed cross-linked structures, but with a different surface appearance, indicating the change to the surface morphology (due to the presence of FLZ), as shown in Figure 5D–F).

### 2.10. Antifungal Activity

#### 2.10.1. Minimum Inhibitory Concentration (MIC) Assay via Resazurin Reduction Technique

**Figure 6:**
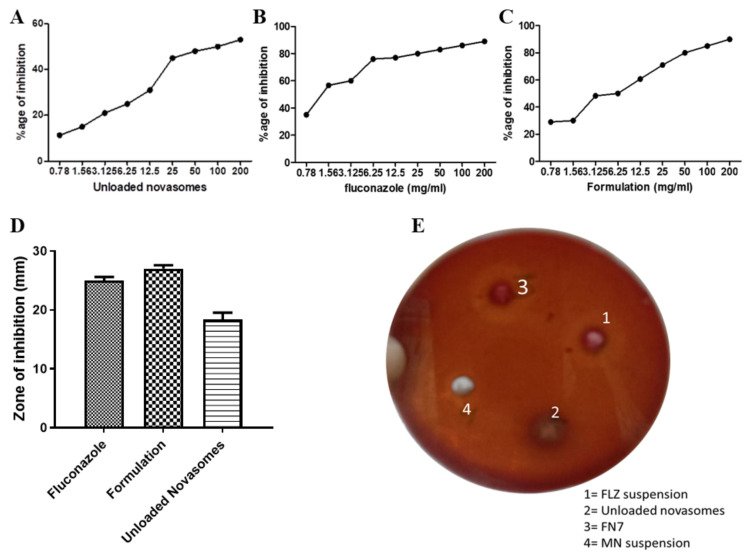
Percentage (%) of inhibition (**A**–**C**) and zone of inhibition (**D**). Values are expressed as mean ± SEM (n = 5), ns = non-significant compared to FLZ suspension. Antifungal activity of FN7, unloaded novasomes, FLZ suspension and standard drug (**E**).

An in vitro antifungal test was performed against *Candida albicans*, as it is the prime causative agent of many superficial and disseminated fungal infections in humans. The in vitro antifungal activity of the FLZ suspension and formulation FN7 is shown in Figure 6. Through checkerboard assay, the MIC was determined at 1.56 mg/mL (*p* = 0.02) for FN7 and 12.5 mg/mL (*p* = 0.001) for the drug-unloaded novasomes. The lower the MIC value, the higher the efficacy of the formulation [12]. Remarkably, the unloaded formulation was also shown to exhibit therapeutic potential against fungal infections, with 31% fungal growth inhibition at a 12.5 mg/mL dose. Oleic acid contains fixed-bend C=C bonds, allowing it to occupy a wider cross-section when entering the fungal membrane. The addition of oleic acid in the formulation assisted the permeation and mobility of the novasomes in the fungal cell membrane, which resulted in the higher antifungal action of the novasomes. Therefore, it was noted that the inclusion of polyunsaturated lipids in the membrane increased oxidative stress, and was thus found to be the cause of the antifungal action of FFAs. FN7 showed lower MIC in comparison to the FLZ suspension, which could be attributed to the high discharge and eventual diffusion of FLZ from FN7, together with the antifungal action of FFAs in comparison with the FLZ suspension.

#### 2.10.2. Agar-Well Diffusion Method

It is noted that the zone of inhibition shown by FN7 was greater than the zones shown by the unloaded novasomes and the FLZ suspension (27 ± 0.577 nm, 18.33 ± 1.202 nm and 25 ± 0.577 nm, respectively). The unloaded novasomes showed remarkable antifungal activity, as shown in Figure 6 D and E. In antifungal activity, the medicated novasomes formulation FN7 showed valuable results against *Candida albicans*, with a maximum zone of inhibition. The FLZ suspension showed less zone of inhibition than the drug-loaded novasomes due to the poor diffusion of FLZ from the suspension.

### 2.11. Effect of Storage Conditions on Nanoparticles

The formulation exposed to light and uncovered showed changed in PS and ZP. The PS was 129 nm, 138 nm and 147 nm after 1, 3 and 6 months, respectively. The ZP was −21, −16 and −09 after 1, 3 and 6 months, respectively. The PS of the FN7 formulation stored in a refrigerator slightly increased with the passage of time. The PS was 110 nm, 111 nm, 113 nm and 115 nm at 0, 1, 3 and 6 months, respectively. The ZP of the stored formulation was measured at intervals of 0, 1, 3 and 6 months. The zeta potential was −24, −22, −19 and −18 at 0, 1, 3 and 6 months, respectively. The percentage release of FLZ from FN7 was 96%, 95.53%, 92.23% and 90.56% at 0, 1, 3 and 6 months, respectively. The formulation placed in a refrigerator was more stable than the formulation placed in sunlight without covering; this is due to the effect of light on the drug and the components of the novasome formulation.

### 2.12. Toxicity Studies of Novasomes

The hematology and biochemical parameters are presented in Table 3 and Table 4. No rats died during the whole period of study. The weight of the rats and their intake of food remained the same, but the water intake of the experimental group increased compared to the control group. The hematological parameters were the same during the application period of the novasomes, but a slight change of lymphocytes in both male and female rats in the recovery phase was observed. A significant (*p* < 0.01) decrease in the number of neutrophils was observed in the recovery phase. In the administration phase, the level of creatinine was slightly lower in female rats. The levels of triglycerides and cholesterol were slightly higher in the administration phase compared to the control group, and in the recovery phase, the cholesterol and triglycerides returned to normal levels in the experimental group [13]. The hematological and biochemical parameters suggested that novasomes are safe, showed no toxic effects and have the potential for use in the topical delivery of antifungal drugs.

## 3. Materials and Methods

### 3.1. Materials

The FLZ was kindly provided by CCL Pharmaceutical Pvt. Ltd., Lahore, Pakistan. The cholesterol was purchased from Alfa Aaeser, Germany. The oleic acid, sodium hydroxide, potassium phosphate monobasic and stearic acid were purchased from Daejung Chemical, Korea. The Span 60, Span 80, dimethyl sulfoxide (DMSO) and Tween 80 were purchased from Sigma-Aldrich, Germany. The ethanol was purchased from Merck, Darmstadt, Germany. All other chemicals and solvents were of analytical grade and were received without further modification.

### 3.2. Preparation of FLZ-Loaded Novasomes

The FLZ-loaded novasomes were prepared by the method of ethyl alcohol injection [14]. Novasomes were developed using different ratios of SAA, FFA, and cholesterol concentrations, as shown in Table 5. A total of 20 mg of FLZ, the selected SAA and FFA and 30 mg of cholesterol were dissolved in ethanol, and the mixture was placed in a water bath at 60 °C for 60 min. A five-times greater volume of phosphate-buffered saline (PBS, pH 7.4) was measured into another beaker, placed on a magnetic stirrer at the same temperature and the previously prepared mixture was injected in the PBS being stirred at 900 rpm. Sudden turbidity was an indication of the preparation of novasomes. After the appearance of turbidity, the preparation was stirred continuously for 2 h for particle-size reduction. All formulations were kept at 4 °C until use [15].

### 3.3. Particle Size and Zeta Potential Analysis

The PS and ZP analysis of different FLZ-loaded novasomes was measured by a Zeta-sizer 2000 (Malvern Instrument Ltd., UK). The novasome formulations were diluted before analysis at room temperature. Each analysis was performed in triplicate and the mean value was recorded.

### 3.4. Percent Entrapment Efficiency (EE)

The EE of the prepared novasomes was determined by indirectly measuring the free amount of FLZ (un-entrapped) [16]. To do so, 1 mL of each novasome formulation was centrifuged at 20,000 rpm for an hour at 4 °C. The clear supernatant was diluted and then FLZ concentration was determined via UV-VIS spectrophotometer at 210 nm. EE% was calculated using this equation:

(1) $$\mathrm{EE}\left(\%\right)\mathrm{of}\;\mathrm{novasoems}=\frac{\mathrm{Total}\;\mathrm{amount}\;\mathrm{of}\;\mathrm{FLZ}-\mathrm{Free}\;\left(\mathrm{unentrapped}\right)\;\mathrm{FLZ}}{\mathrm{Total}\;\mathrm{amount}\;\mathrm{of}\;\mathrm{FLZ}}\;\times 100$$

### 3.5. In Vitro Drug Release

The release of FLZ from different FLZ-loaded novasomes was determined by performing a release study using the USP Dissolution test apparatus II (paddle apparatus) with a dialysis membrane (cellulose membrane), which was presoaked overnight in PBS (pH 7.4). A constant volume of each of the FLZ-loaded novasomes (equivalent to 2 mg of FLZ) was placed in different glass tubes with a diameter of 2.5 cm and a length of 7.5 cm. A cellulose membrane was used to cover the glass tubes at one end, and the other end was attached to the shaft. The shafts were then lowered into beakers containing 500 mL of phosphate buffer saline of pH 7.4 as a dissolution medium. The temperature of the dissolution medium was kept constant at 37 °C using a water jacket containing water, onto which the beakers containing the dissolution medium were mounted. The shafts in the beakers were adjusted to a rotation of 100 rpm and the beakers were covered throughout the experiment to prevent evaporation of the dissolution medium. The samples were withdrawn at 0.5, 1, 2, 3, 4, 5, 6, 7 and 8 h and analyzed at 210 nm using a UV-visible spectrophotometer.

### 3.6. Release Kinetics

The release of the drug from the formulation plays an important role when the focus is on sustained release or controlled release. It is crucial to investigate the release pattern of the drug from the formulation because it governs the efficacy of the formulation. Among the different models used to study the release kinetics, zero-order kinetics, first-order kinetics, the Higuchi model, the Korsmeyer–Peppas model and the Hixon–Crowell model are used here.

### 3.7. Fourier-Transform Infrared Spectroscopy (FTIR)

The FTIR spectra of the FLZ, oleic acid, Span 60, cholesterol, drug-unloaded and drug-loaded novasomes were determined using an attenuated total reflectance FTIR spectrophotometer (Bruker, Alpha, Germany), using a 5 mg quantity of each sample. This approach was used to analyze the chain interaction between the excipients and the drug; 20 scans were captured for each spectrum between 4000 and 800 cm^−1^ wave number with a resolution of 10 cm^−1^.

### 3.8. Differential Scanning Calorimetry (DSC)

The thermal characterization of the novasomes was performed by differential scanning calorimeter (DSC-60 Shimadzu, Germany) in order to assess the thermal stability of the FLZ-loaded and unloaded novasomes. The 5 mg sample of FLZ, excipients and novasomes were heated from 0 to 400 °C at a heating rate of 10 °C/min.

### 3.9. Thermogravimetric Analysis (TGA)

Thermogravimetric analysis is used to examine the stability of novasomes at high temperatures and the fraction of volatile components by monitoring any weight loss that occurs when the novasomes are heated at a constant rate. The 5 mg sample of FLZ, excipients and novasomes were heated from 0 to 400 °C at a heating rate of 10 °C/min.

### 3.10. Scanning Electron Microscopy (SEM)

The surface morphology of the developed novasomes (drug-unloaded and loaded) was observed using a scanning electron microscope (JEOL-6700F).

### 3.11. Antifungal Activity

#### 3.11.1. Minimum Inhibitory Concentration (MIC) Assay via Resazurin Reduction Technique

The MIC value is the lowest concentration of a prepared formulation that can completely inhibit the growth of *Candida albicans*. The micro-well dilution method [17] was used to determine the MIC for the prepared novasomes. Microdilution and the agar-dilution method are usually used to determine the MIC values, but the agar-dilution method is lengthy, time consuming and less accurate. Conversely, the microdilution method is consistent, precise, inexpensive and easy to perform. The microdilution method is improved by the use of resazurin dye as a redox indicator. The inocula were primed and their concentrations maintained at 10^6^ CFU/mL. The FN7 optimum novasomes loaded with FLZ, unloaded novasomes and FLZ as standard drug were all diluted twofold by DMSO in a 96-well plate [18]. A total of 40 µL of brain–heart infusion (the growth medium) was filled in each well, and then 10 µL inoculum was added along with 50 µL diluted formulae. For the negative control, we used DMSO, and the plates were then incubated at 37 °C for 24 h, after which 10 µL of resazurin dye was added to each well, which was then incubated again in the dark for an hour at 37 °C. Any change in color as a result of the dye was calculated at 492 nm via the microtiter plate reader (Tecan Sunrise absorbance reader, UK) [19]. The one-way ANOVA was used as statistical tool to evaluate the results. The percentage of inhibition was obtained as follows:

(2) $$\mathrm{Percentage}\;\mathrm{of}\;\mathrm{inhibition}\;=1-\left(\frac{\mathrm{mean}\;\mathrm{of}\;\mathrm{test}\;\mathrm{wells}}{\mathrm{mean}\;\mathrm{of}\;\mathrm{control}\;\mathrm{wells}}\right)\times \;100$$

#### 3.11.2. Agar-Well Diffusion Method

The agar-well diffusion method was used to determine the antifungal activity of the FLZ-loaded FN7 novasome formulation. A MacConkey agar plate surface was inoculated with *Candida albicans* using the streak plate method. MacConkey agar is a selective and differential growth medium that retards the growth of Gram-positive bacteria due to crystal violet and bile salts, as the *Candida albicans* clinical specimens we used may also contain bacteria. Therefore, we used selective media for yeast *Candida albicans* growth. A sterile cork-borer was used to punch a hole of 6–8 mm diameter, and a volume of 20 µL of each of the optimum novasomes (FN7), FLZ suspension (standard drug), and unloaded novasomes was added into each well. The agar plates were then incubated for 48 h [20,21].

### 3.12. Effect of Storage Conditions on Novasomes

The best selected formulation was divided into three parts and placed in three vials (15 mL each). Two flasks were covered with aluminum foil and placed in refrigerator at −4 °C, and one sample was placed at room temperature without a covering and exposed to daylight. The particle size, zeta potential measurements and in vitro drug release of the FLZ were made immediately and after 1, 3 and 6 months [22].

### 3.13. Toxicity Studies of Novasomes

A toxicity study [13] was performed on albino rats for a period of 90 days. A total of 24 rats were selected for the study and the hair on the back of the rats was removed every 7 days. The duration of the administration and recovery phases was 90 and 16 days, respectively. The FN7 formulation was applied onto the skin (10% body surface area) and washed with sterile water every 6 h. Any change in food and water intake was observed and body weight changes were also observed. After 90 days, the rats were fasted for 18 h and ketamine was used to anesthetize the rats. Samples of blood were withdrawn from the abdominal artery, centrifuged at 4000 rpm for 15 min and analyzed for hematology and biochemical parameters. The observed hematological parameters were hemoglobin, RBCs, WBCs and platelet count. The biochemical parameters were the level of albumin, globulin, creatinine, glucose, cholesterol and triglycerides. The Student t test was used as a statistical tool to evaluate the results.

## 4. Conclusions

Novasomes of FLZ were prepared successfully using the ethanol injection method, using SAAs and FFAs in a ratio of 2:1 (*w*/*v*) with 30 mg of cholesterol. The developed dosage form successfully delivered the antifungal drug to the target site for a prolonged period of time. MIC assay and zone of inhibition studies, with the help of the agar-well method, showed that the formulation has a good antifungal effect and the toxicity studies showed that the novasome formulations are non-toxic and safe to use. Thus, novasomes can be said to be a promising and effective system to encapsulate the drug and can also be used for the treatment of topical fungal infections. The novasomes sustained the release of the drug and can therefore be effectively used for the management of fungal infections of the skin. In the future, we plan to study these novasomes using different fatty acids as well as different antifungal agents for exploratory clinical experimentation.

## Figures and Tables

**Table 1:** PS, PDI, ZP and EE of all novasome formulations.

Code	PS (nm)	PDI	ZP (mV)	EE (%)
FN1	145	0.120	−22	76.34
FN2	178	0.230	−19	70.23
FN3	123	0.345	−23	56.12
FN4	149	0.319	−17	80.20
FN5	236	0.287	−20	45.09
FN6	298	0.067	−18	89.18
FN7	110	0.023	−24	94.45
FN8	120	0.069	−21	49.82
FN9	141	0.267	−21	52.98
FN10	192	0.345	−20	90.06

**Table 2:** Release kinetics by different models.

Code	Zero Order	First Order	Higuchi	Korsmeyer–Peppas		Hixon–Crowell
	R^2^	R^2^	R^2^	R^2^	n	R^2^
FN1	0.9878	0.9581	0.831	0.993	1.141	0.9693
FN2	0.9935	0.9599	0.8456	0.9959	1.091	0.9737
FN3	0.9961	0.984	0.8497	0.9974	1.066	0.9893
FN4	0.9967	0.9898	0.8859	0.9973	0.958	0.9938
FN5	0.9888	0.9439	0.8285	0.9948	1.152	0.9612
FN6	0.9898	0.9797	0.892	0.9925	0.916	0.9884
FN7	0.978	0.9666	0.9356	0.9975	0.793	0.9834
FN8	0.9805	0.9818	0.9308	0.9978	0.806	0.9931
FN9	0.9903	0.9656	0.8668	0.9903	0.995	0.9773
FN10	0.9984	0.9685	0.8809	0.9987	0.973	0.9836

**Table 3:** Hematological parameters (n = 12).

Phases	Hemoglobin g/dL	Red Blood Cells (RBCs) (10^3^/µL)	White Blood Cells (WBCs) (10^6^/µL)	Platelet Count (10^3^/µL)
Control group				
Administration	14.2 ± 1.35 **	7.99 ± 2.19 **	5.27 ± 1.80 *	1023 ± 3.45 **
Recovery	14.3 ± 1.94 *	8.01 ± 2.31 *	5.29 ± 1.67 *	1022 ± 3.28 **
Experimental group				
Administration	14.3 ± 1.18 **	7.96 ± 2.17 **	5.26 ± 1.21 **	1019 ± 3.29 *
Recovery	14.4 ± 1.03 *	8.21 ± 2.11 *	5.28 ± 1.39 *	1020 ± 3.12 **

* < 0.05 and ** < 0.01.

**Table 4:** Biochemical parameters (n = 12).

Phases	Albumin (g/dL)	Globulin (mg/dL)	Creatinine (mg/dL)	Glucose (mg/dL)	Cholesterol (mg/dL)	Triglycerides (mg/dL)
Control group						
Administration	2.3 ± 0.23 *	157 ± 2.15 **	0.60 ± 0.04 *	161 ± 3.78 **	56 ± 1.32 **	38 ± 1.02 **
Recovery	2.4 ± 0.21 *	156 ± 2.65 **	0.58 ± 0.05 *	158 ± 4.09 *	55 ± 1.54 **	37 ± 1.11 *
Experimental group						
Administration	2.2 ± 0.67 **	158 ± 2.98 *	0.58 ± 0.07 **	163 ± 4.01 *	63 ± 1.76 **	42 ± 1.20 *
Recovery	2.5 ± 0.45 *	160 ± 2.56 *	0.56 ± 0.06 *	160 ± 3.21 **	54 ± 1.96 *	36 ± 1.32 *

* < 0.05 and ** < 0.01.

**Table 5:** Composition of different novasome formulations.

Formulation	Type of SAA	Type of FFA	Ratio of SAA: FFA	Cholesterol (mg)
FN1	Span 80	Oleic acid	2:1	30
FN2	Span 80	Oleic acid	1:1	30
FN3	Span 80	Stearic acid	2:1	30
FN4	Span 80	Stearic acid	1:1	30
FN5	Tween 80	Oleic acid	2:1	30
FN6	Tween 80	Oleic acid	1:1	30
FN7	Span 60	Oleic acid	2:1	30
FN8	Span 60	Oleic acid	1:1	30
FN9	Span 60	Stearic acid	2:1	30
FN10	Span 60	Stearic acid	1:1	30

## Data Availability

Not applicable.

## Sample Availability

Samples of the compounds are available from the authors.
